# Tobacco Rattle Virus Vector: A Rapid and Transient Means of Silencing *Manduca sexta* Genes by Plant Mediated RNA Interference

**DOI:** 10.1371/journal.pone.0031347

**Published:** 2012-02-01

**Authors:** Pavan Kumar, Sagar Subhash Pandit, Ian T. Baldwin

**Affiliations:** Department of Molecular Ecology, Max Planck Institute for Chemical Ecology, Jena, Germany; Centro de Pesquisas René Rachou, Brazil

## Abstract

**Background:**

RNAi can be achieved in insect herbivores by feeding them host plants stably transformed to express double stranded RNA (dsRNA) of selected midgut-expressed genes. However, the development of stably transformed plants is a slow and laborious process and here we developed a rapid, reliable and transient method. We used viral vectors to produce dsRNA in the host plant *Nicotiana attenuata* to transiently silence midgut genes of the plant's lepidopteran specialist herbivore, *Manduca sexta*. To compare the efficacy of longer, undiced dsRNA for insect gene silencing, we silenced *N. attenuata*'s dicer genes (*Na*DCL1- 4) in all combinations in a plant stably transformed to express dsRNA targeting an insect gene.

**Methodology/Principal Findings:**

Stable transgenic *N. attenuata* plants harboring a 312 bp fragment of *Ms*CYP6B46 in an inverted repeat orientation (*ir*-CYP6B46) were generated to produce CYP6B46 dsRNA. After consuming these plants, transcripts of CYP6B46 were significantly reduced in *M. sexta* larval midguts. The same 312 bp cDNA was cloned in an antisense orientation into a TRV vector and *Agro*-infiltrated into *N. attenuata* plants. When larvae ingested these plants, similar reductions in CYP6B46 transcripts were observed without reducing transcripts of the most closely related *Ms*CYP6B45. We used this transient method to rapidly silence the expression of two additional midgut-expressed *Ms*CYPs. CYP6B46 transcripts were further reduced in midguts, when the larvae fed on *ir*-CYP6B46 plants transiently silenced for two combinations of *Na*DCLs (DCL1/3/4 and DCL2/3/4) and contained higher concentrations of longer, undiced CYP6B46 dsRNA.

**Conclusions:**

Both stable and transient expression of CYP6B46 dsRNA in host plants provides a specific and robust means of silencing this gene in *M. sexta* larvae, but the transient system is better suited for high throughput analyses. Transiently silencing *Na*DCLs in *ir*-CYP6B46 plants increased the silencing of *Ms*CYP6B46, suggested that insect's RNAi machinery is more efficient with longer lengths of ingested dsRNA.

## Introduction

RNA interference (RNAi), the double stranded RNA (dsRNA) mediated gene silencing was discovered in nematodes in 1998 [1]. During the RNAi process, dsRNA produced in the nucleus is transported to the cytoplasm; alternatively, exogenous dsRNA can be taken up by cells with the help of the cell surface protein, SID [from systemic RNAi deficient mutants (*sid*)] [2]. In the cytoplasm, dsRNA is cleaved by RNaseIII type enzymes (dicers) to produce approximately 22 bp fragments, called small interfering RNAs (siRNAs) [3], [4]. One strand of the siRNA (guide strand) is incorporated into the RNA-induced silencing complex (RISC) with the perfectly complementary site in a target mRNA to form a guide strand-target mRNA duplex [5]. The target mRNA is then sliced by the Argonaute protein of RISC [6]. In plants and nematodes, RNAi is amplified by the activity of RNA-dependent RNA polymerases (RdRPs). This enzyme extends the guide strand that is bound to the target mRNA towards its 5′ end [3], [7]. The long dsRNA formed in this process re-enters the RNAi cycle after it is cleaved by dicers. RNAi-mediated gene silencing becomes a systemic process as the siRNAs spread to neighboring cells to induce a fresh cycle of dicing and splicing Mlotshwa1 [3], [7], [8].

This endogenous gene silencing mechanism has been exploited as a reverse genetic tool for several model organisms and has also been proposed as a potentially useful tool for pest control [9], [10]. However, there are two major limitations to the widespread use of RNAi in insects: 1) the generation of stably transformed lines of insects for the reverse genetics research is onerous, and 2) the apparent lack of key components of the RNAi pathway in insects, namely SIDs and RdRPs [11], which requires that large quantities of triggering siRNAs be continuously administered to sustain gene silencing. To understand if RNAi is functional in insects, researchers have delivered dsRNA to insects by various methods: feeding [12], [13], [14], [15], [16], [17], injection [18] and exogenous application [19]. Interestingly, the success of silencing genes by these different delivery modes differs amongst the various insect orders: Coleoptera [9], [20], Diptera [21], Hemiptera [13], [14], Hymenoptera [22], Isoptera [23], Lepidoptera [12], [24] and Orthoptera [25]. As RdRP orthologs are thought to be absent in most orders of the insects [11], the spontaneous amplification of RNAi is considered unlikely and the silencing effects are thought to be transient. Hence, sustained RNAi would require a continuous input of large quantities of dsRNA [13], [15], [17]. This limitation of the RNAi procedures could be alleviated in the case of herbivorous insects if the insects' host plants could be transformed to express dsRNA targeting insect genes. This approach has been shown to be effective by several researchers [13], [14], [15], [17]. Their work revealed that when insects feed on dsRNA-producing transgenic host plants, dsRNA molecules penetrate the herbivores' gut cells and reduce the expression of the target gene by post transcriptional gene silencing. This strategy, which recommended the use of stably transformed plants for achieving gene silencing in herbivorous insects was called plant mediated RNAi (in this paper abbreviated PMRi).

RNAi and PMRi in insects provide a new reverse genetics research tool which has the potential to enable ‘real time’ analysis of insect gene function during herbivorous insect-plant interactions, as well as a powerful new means of controlling pests. However, the process appears to be challenging to implement for one of the most specious orders of herbivorous insects, the Lepidoptera [24], and is constrained by the time and labor requirements of generating stably transformed plants. Given that microarray-based transcriptomics of insects feeding on plants have revealed a plethora of regulated genes in insect exomes, more high throughput means of PMRi are required. Pitino and colleagues suggested a transient transformation strategy against aphids based on the *Agro*-infiltration of *Nicotiana benthamiana* leaf discs, [14]; whereas, a plant-virus based RNAi technique with these qualities was suggested against nematodes [26], [27]. Dubreuil *et al.* [26] and Valentine *et al.* [27] engineered the tobacco rattle virus (TRV) for dsRNA production in plants. These researchers transiently transformed *N. benthamiana* plants with viral-based constructs and demonstrated RNAi in nematodes feeding on these plants. Here we report the development of a similar plant-virus based dsRNA producing system (VDPS) for the silencing of lepidopteran genes.

To examine the utility of VDPS against insects, we attempted to silence genes of the herbivorous insect, *Manduca sexta* (Sphingidae, Lepidoptera), through its native host plant, *N. attenuata*. This plant-herbivore system was chosen because stable as well as TRV-based transient transformation systems are well established for *N. attenuata* [28], [29] and the trophic delivery of dsRNA has recently been reported for *M. sexta* [16]. Secondly, unlike *Bombyx mori* or *Helicoverpa armigera* which represent domesticated and pest insect models, respectively, *M. sexta* is an ecological insect model whose interactions with its host are well characterized. Therefore, the development of a VDPS for *M. sexta* would be a useful tool for the study of ecological interactions. As the first gene targets for silencing, we selected three midgut expressed cytochrome P450 (CYP) genes. We compared the silencing efficiency of stable PMRi and the new transient VDPS, for one of the candidate CYPs. Second, Terenius *et al.* stated that, “it is always a concern that based on the mechanism of gene silencing, RNAi treatments may in some cases induce off-target effects” [24]; considering this possibility, we examined the specificity of VDPS for the silencing of *M. sexta* genes and analyzed “off-target” effects on the expression of CYPs that share the highest sequence identity with the three targeted CYPs. In addition, we silenced *N. attenuata*'s four dicer (DCL) genes during PMRi to evaluate if ingestion of longer undiced dsRNA increases the silencing efficiency of the targeted insect genes.

## Results

### Selection of *Ms*CYPs for silencing test

In preparation for a more detailed analysis of *M. sexta* larvae's remarkable ability to tolerate dietary nicotine, we found literature that reported the transcripts of three *Ms*CYPs (CYP6B46, CYP4M1 and CYP4M3) to be up-regulated in response to dietary nicotine intake [30], [31].

**Figure 1:**
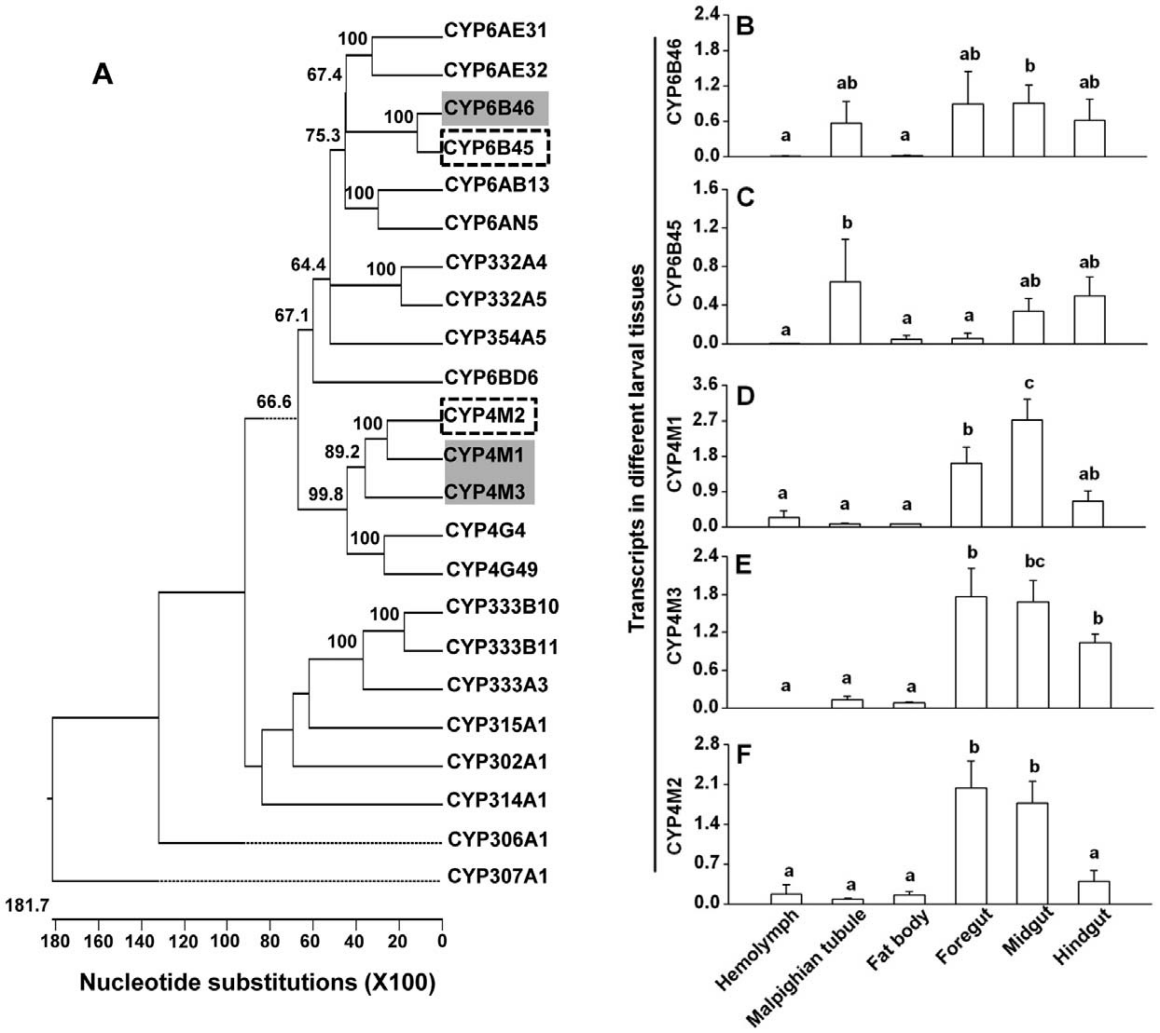
Selection of *M. sexta* CYPs for plant mediated RNAi (PMRi) and their spatial expression profiles. (**A**) Phylogenetic relationship among *M. sexta* CYPs (complete ORFs) as calculated by the Clustal-W program. CYPs selected for silencing by PMRi are shaded in gray and their most closely related CYPs that were analyzed for off-target co-silencing are in dashed boxes. Thousand bootstrapping trials were conducted (only the bootstrap values >50 displayed). Transcript levels (relative to ubiquitin) of (**B**) CYP6B46, (**C**) CYP6B45, (**D**) CYP4M1, (**E**) CYP4M3 and (**F**) CYP4M2 in hemolymph, Malpighian tubules, fat body, foregut, midgut and hindgut of 5^th^ instar *M. sexta* larvae feeding on *N. attenuata* (WT) plants. Bars labeled with different letters indicate the significant differences as determined by one way ANOVAs (*p*≤0.05).

To identify the CYPs that were most closely related to the three target candidates CYP6B46, CYP4M1 or CYP4M3, and hence potentially at the risk of being co-silenced by the RNAi procedure, we aligned all 23 *Ms*CYP nucleotide sequences that were available as complete coding sequences in the NCBI database to construct a phylogenetic tree (Fig. 1A). We found that CYP6B45 had the highest sequence similarity to CYP6B46 (80.2%), whereas CYP4M2 was similar to both CYP4M1 (63.5%) and CYP4M3 (55.2%). Notably, CYP4M1 and CYP4M3 shared 53.3% sequence identity with each other. Four homologous regions of >21nt were identified in the alignment of CYP6B46 and CYP6B45 that were exactly identical (+231 to +267, +415 to +437, +1306 to +1334 and +1384 to +1430). No homologous regions of >21nt identical bases could be identified in the alignments of CYP4M1, CYP4M2 and CYP4M3.

CYP6B46, CYP4M1 and CYP4M3 had ORFs of comparable lengths (1524, 1515 and 1503 bp, respectively). In order to keep the length of the undiced dsRNA precursor uniform for all three genes we cloned the cDNA fragments of ≥300 bp from each of these genes into the VDPS vector. To accomplish this, we analyzed these sequences for the availability of primer binding sites ≥300 bp apart from each other and selected regions to be cloned in each candidate cDNA. The similarity of this selected ≥300 bp region from CYP6B46 with its homolog in CYP6B45 was 80.4%. This region contained one >21nt (+415 to +437 of the ORF =  +112 to +134 of selected ≥300 bp fragment) stretch that was identical in the two aligned fragments (Fig. S1A). The ≥300 bp regions selected from CYP4M1 and CYP4M3 were homologous to each other (54.1% similar), whereas their similarity with the homologous region from CYP4M2 was 64.5% and 57.1%, respectively (Fig. S1B). The exact sizes of these selected regions were 312 bp (+301 to +612), 338 bp (+1000 to +1337) and 322 bp (+966 to +1287) in CYP6B46, CYP4M1 and CYP4M3, respectively.

PMRi is thought to mainly target genes that are expressed in gut tissues [13], [15], [17]. Therefore, to ascertain whether the candidate genes (CYP6B46, CYP4M1 and CYP4M3) were gut expressed, we profiled their transcripts along with the transcripts of the allied co-target (CYP6B45 and CYP4M2) genes in hemolymph, Malpighian tubules, fat body, foregut, midgut and hindgut. All five genes were found to have relatively higher expression levels in the gut regions as compared to the other tissues (*p*≤0.05; Fig. 1B). The primers used for this profiling specifically amplified respective insect cDNA; they did not produce any amplicons when plant cDNA was used as a PCR template (Fig. S1C).

### Stable transgenic plant mediated RNAi (PMRi) for *Ms*CYP6B46

**Figure 2:**
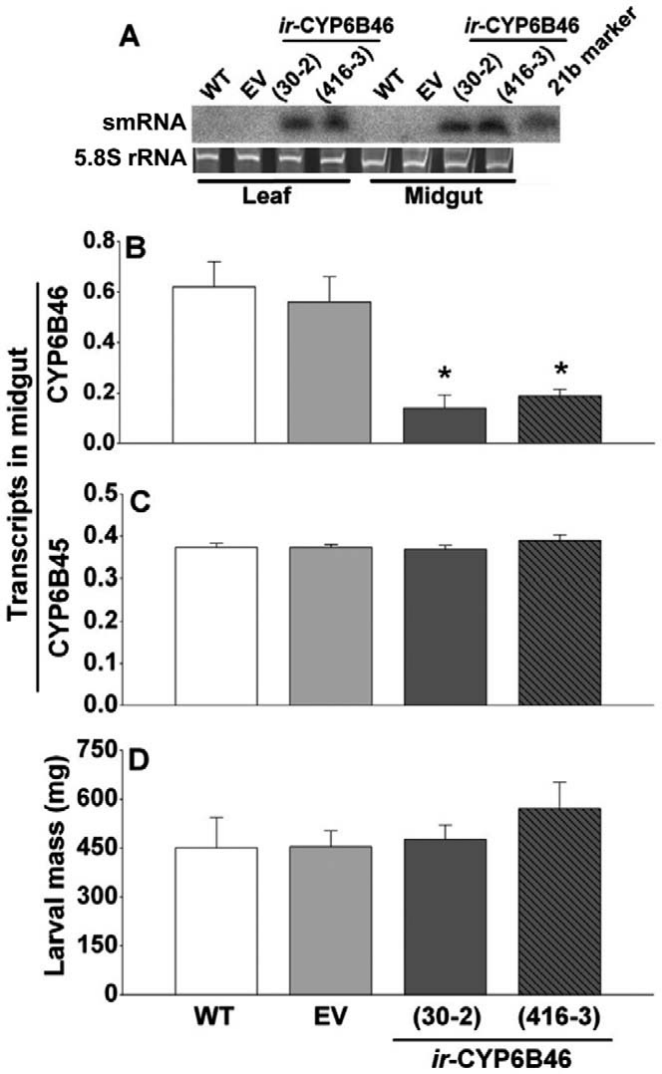
PMRi of *M. sexta* CYP6B46 using stably transformed *N. attenuata* plants. (**A**) Northern hybridizations revealed the presence of small RNAs of CYP6B46 in the leaves of two independent lines of stably transformed plants, *ir*-CYP6B46 (30-2) and *ir-*CYP6B46 (416-3), and in the midguts of 4^th^ instar larvae feeding on these plants. RNA samples from leaves of WT and EV (empty vector transformed stable line) plants and from the midguts of larvae feeding on WT and EV leaves were used as negative controls. Similar fluorescence intensity of the ethidium bromide stained 5.8 S rRNA bands reflected the equal loading of LMW RNA. Low molecular weight RNA from leaf or midgut loaded on the gel in each lane was a pool of three biological replicates. smRNA length of 21 b denoted by marker. Transcript abundance (relative to ubiquitin) of: (**B**) CYP6B46 (target gene) and closely related (**C**) CYP6B45 (off-target) in the midguts of 4^th^ instar larvae. (**D**) Larval mass of 4^th^ instar larvae feeding on WT, EV, *ir*-CYP6B46 (30-2) and *ir-*CYP6B46 (416-3) *N. attenuata* plants for 14 days. Asterisk indicates the significant differences as determined by one way ANOVAs (*p*≤0.05).

We generated stable transgenic lines of *N. attenuata* plants transformed with a pSOL8 vector harboring an inverted repeat (*ir*) of the selected 312 bp CYP6B46 cDNA fragment (Fig. S2A). A single insertion of the transgene was confirmed by southern hybridization, in two T_2_ generation lines (*ir-*CYP6B46 30-2 and *ir-*CYP6B46 416-3) that were generated from two independent transformation events (Fig. S2B). Transcription of 312 bp CYP6B46 ‘ir’ insertions and the subsequent formation of diced small dsRNA (21–24nt) (smRNA) in the leaves of the *ir-*CYP6B46 lines were ascertained by northern hybridization (Fig. 2A). CYP6B46 smRNA was not detected in the leaves of controls wild type (WT) and empty pSOL8 vector (EV) plants. Morphology and development of the plants of both *ir-*CYP6B46 lines were similar to those of WT or EV plants.

Freshly hatched neonates of *M. sexta* larvae were placed on control and *ir-*CYP6B46 (30-2 and 416-3) lines and allowed to feed freely for 14 days. Using Northern hybridization, we confirmed the presence of CYP6B46 smRNA in their midgut (Fig. 2A). No CYP6B46 smRNA could be detected in the midguts of larvae that fed on the WT and EV plants (Fig. 2A). Further, CYP6B46 transcript levels in the midgut of these 14 d old larvae feeding on two *ir-*CYP lines were significantly reduced (three fold) compared to those of the larvae feeding on WT or EV plants (*p*≤0.05; Fig. 2B). Interestingly, the silencing of this gene had no effect on larval mass gain recorded after 14 d of feeding (*p*>0.05; Fig. 2D).

### PMRi mediated silencing was target gene specific

To ascertain if the silencing was specific to the target gene we quantified the transcript levels of the closely related off-target gene, *CYP6B45*. The transcript levels of *CYP6B45* in the midguts of larvae feeding on WT, EV and transgenic *ir-*CYP6B46 (30-2 and 416-3) plants did not differ (*p*>0.05; Fig. 2C).

### VDPS mediated silencing of *Ms*CYP6B46, *Ms*CYP4M1 and *Ms*CYP4M3

**Figure 3:**
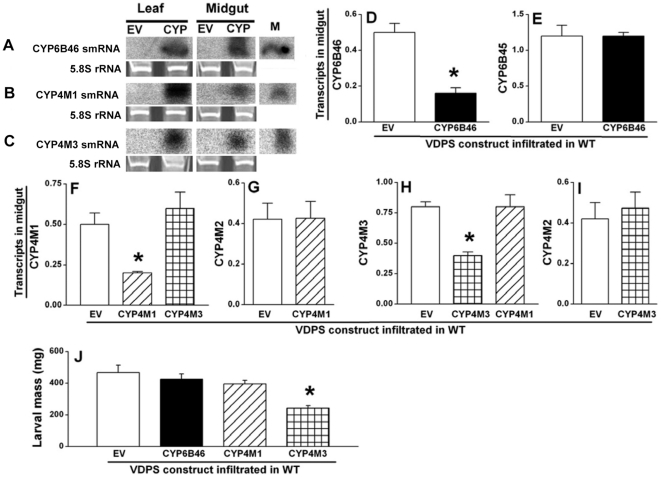
Efficiency and specificity of CYP6B46, CYP4M1 and CYP4M3 silencing by viral dsRNA-producing system (VDPS). Northern hybridization showing the smRNAs of (**A**) CYP6B46 (**B**) CYP4M1 and (**C**) CYP4M3 in WT leaves infiltrated with VDPS-EV, -CYP6B46, -CYP4M1 and -CYP4M3 constructs, respectively, as well as in the midguts of 4^th^ instar larvae feeding on the leaves of plants inoculated with the respective constructs. Lane M shows 21 b oligonucleotide that was used as size marker as well as a positive control for hybridization. Similar fluorescence intensity of the ethidium bromide stained 5.8 S rRNA bands reflected the equal loading of LMW RNA. Transcript abundance (relative to ubiquitin) of the target genes. (**D**) CYP6B46 in the midguts of 4^th^ instar larvae feeding on VDPS-EV and -CYP6B46, (**F**) CYP4M1 in the midguts of 4^th^ instar larvae feeding on VDPS-EV, -CYP4M1 and -CYP4M3, and (**H**) CYP4M3 in the midguts of 4^th^ instar larvae feeding on VDPS-EV, -CYP4M3 and -CYP4M1 plants, respectively. Transcript abundance (relative to ubiquitin) of closely related, off-target genes (**E**) CYP6B45 in the midguts of 4^th^ instar larvae feeding on VDPS-EV and -CYP6B46, (**G**) CYP4M2 in the midguts of 4^th^ instar larvae feeding on VDPS-EV and -CYP4M1, and (**I**) CYP4M2 in the midguts of 4^th^ instar larvae feeding on VDPS-EV and -CYP4M3 plants, respectively. (**J**) Mass of 4^th^ instar *M. sexta* larvae fed for 14 days on VDPS-EV, -CYP6B46, -CYP4M1 and -CYP4M3 plants. Asterisk indicates the significant differences as determined by one way ANOVAs (*p*≤0.05).

In order to rapidly and efficiently synthesize the dsRNA *in planta*, virus induced gene silencing (VIGS) vector pTV containing the antisense cDNA fragment (≥300 bp) of *Ms*CYP6B46, *Ms*CYP4M1 or *Ms*CYP4M3 (Fig. S3A) was *Agro*-infiltrated into *N. attenuata* WT leaves, as described by Saedler and Baldwin [29]. The smRNA produced in the leaves was detected by Northern hybridization, using the appropriate gene specific probe (Fig. 3A–3C). For all three CYPs, the negative control leaves (EV) did not contain any of the target smRNAs.

Freshly hatched *M. sexta* neonates were transferred to VDPS-CYP6B46, -CYP4M1, -CYP4M3 and -EV plants. After 14 d of feeding, the midguts of these larvae were analyzed for the presence of the respective smRNAs by Northern hybridization. The expected smRNAs were detected in the midguts of larvae feeding on VDPS-CYP6B46, -CYP4M1 and -CYP4M3 lines (Fig. 3A–3C). The success of the silencing of the target gene was quantified by the reduction in the transcript levels of the target gene in the midguts of larvae feeding on the respective VDPS-CYP line, compared to larvae feeding on VDPS-EV. VPDS reduced CYP6B46 transcripts by three fold (Fig. 3D), reductions that were equivalent to that mediated by PMRi (Fig. 2B). Transcripts of CYP4M1 and CYP4M3 were reduced by 50% in the midguts of larvae feeding on the respective VDPS plants, as compared to that in the midguts of EV fed larvae (*p*≤0.05; Fig. 3F, 3H). Larval performance measured in terms of body mass after feeding for 14 d on VDPS-CYP6B46 and VDPS-EV plants was unchanged (*p*>0.05; Fig. 3J): a result congruent with the larval mass gain observed on stable PMRi lines (*ir*-CYP6B46 and EV) (Fig. 2D). Larvae feeding on VDPS-CYP4M1 plants also did not show any difference in body mass as compared to that of larvae feeding on control EV plants (*p*>0.05; Fig. 3J). However, the larvae feeding on VDPS-CYP4M3 plants gained significantly less mass compared to EV fed larvae (*p*≤0.05; Fig. 3J).

### VDPS mediated silencing is target gene specific

Levels of CYP6B45 transcripts, the off-target gene, remained unchanged in the midguts of larvae feeding on EV or VDPS-CYP6B46 plants (*p*>0.05; Fig. 3E), suggesting that the CYP6B46 silencing attained by VDPS was comparably specific to that attained by stable PMRi (Fig. 2C). Similarly, the silencing of CYP4M1 did not cause co-silencing of CYP4M3 and *vice versa* (*p*>0.05) (Fig. 3F, 3H); moreover, larvae feeding on these two VDPS lines (-CYP4M1 and -CYP4M3) had similar CYP4M2 transcript levels in their midguts (compared to larvae feeding on EV; *p*>0.05) (Fig. 3G, 3I).

### Silencing of plant dicers enhances the silencing of CYP6B46 by PMRi

**Figure 4:**
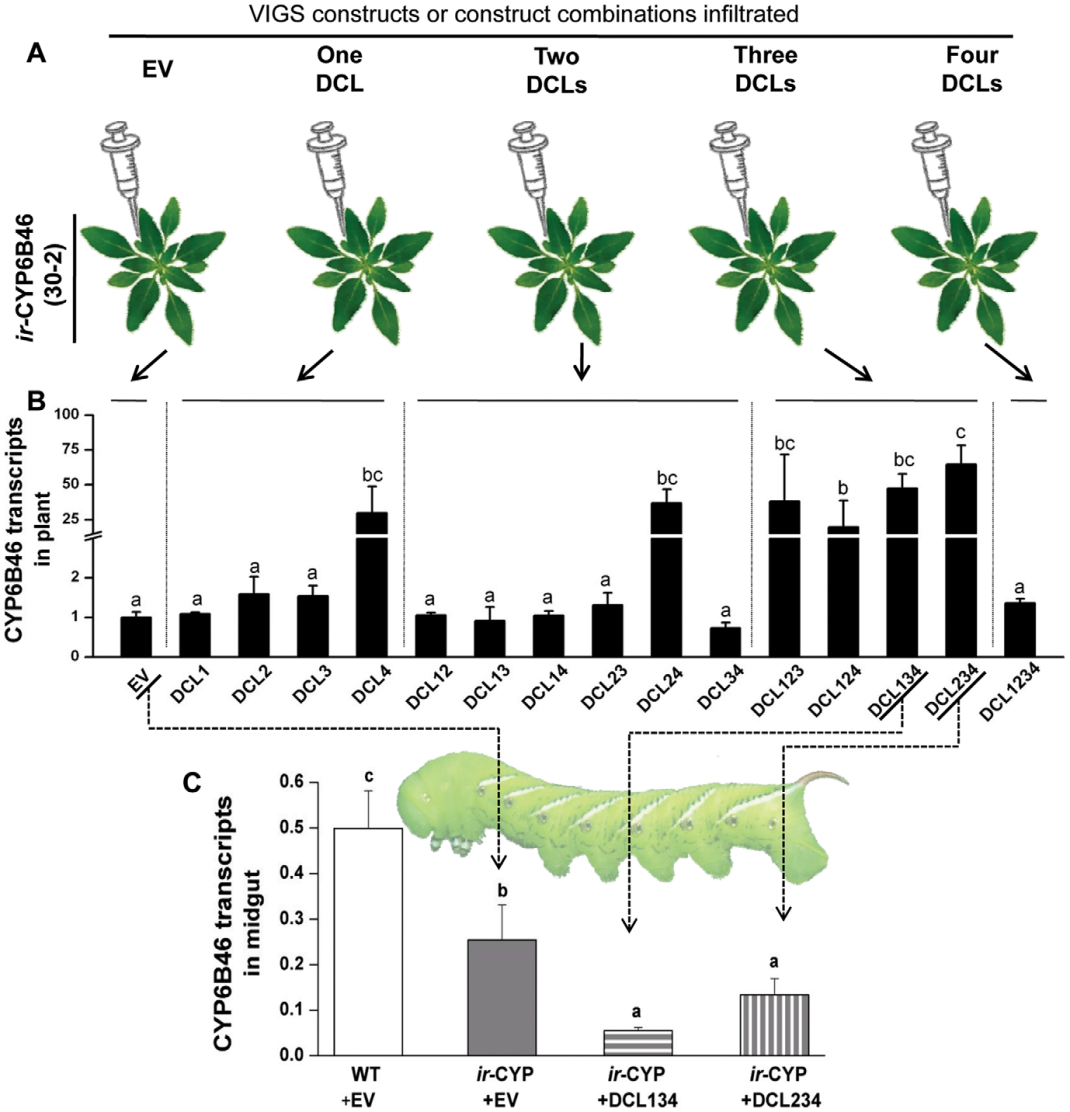
PMRi efficiency is increased after silencing *N. attenuata*'s dicer-like (DCL) genes. (**A**) Schematic representation of the silencing of *N. attenuata*'s four DCLs by virus induced gene silencing (VIGS), in *ir-*CYP6B46 (30-2) stably-transformed plants. *ir*-CYP (30-2) plants were *Agro*-infiltrated with pTVDCL harboring cultures (individually, and in all combinations of DCL1, DCL2, DCL3 and DCL4); pTV (EV) was used as a control. (**B**) Abundance, relative to *Na*Actin, of a 102 bp region of the 5′ end of the 312 bp *Ms*CYP6B46 fragment that the *ir*-CYP (30-2) *N. attenuata* plants were harboring in their genome. The plants had been previously *Agro*-infiltrated with EV or all combinations of vectors designed to silence the expression of the four *Na*DCLs. (**C**) Transcript abundance of CYP6B46 (relative to ubiquitin) in the midguts of 4^th^ instar *M. sexta* larvae, when fed *N. attenuata* leaves containing no *Ms*CYP6B46 dsRNA (WT+EV), small (diced) *Ms*CYP6B46 dsRNA (*ir-*CYP6B46+EV) and on leaves of plants containing higher concentration of longer (102 bp detected by qPCR) *Ms*CYP6B46 dsRNA fragments (*ir-*CYP6B46+ DCL134 and *ir-*CYP6B46+ DCL234). See Fig. S4A and Table S1 for the design of the primers used in the transcript quantification. Bars labeled with different letters indicate significant differences as determined by one way ANOVAs (*p*≤0.05).

DCLs are involved in the biogenesis of smRNA by cleaving longer dsRNA. Four different types of DCLs are reported in higher plants. Their function has been found to overlap in plants, suggesting that one DCL can contribute to and/or compensate for the function of the others. Hence, more than one DCL might be involved in processing long dsRNA [32]. Efficiency of plant mediated silencing of *H. armigera* gene was previously shown to be increased by feeding long dsRNA that was obtained by silencing three Arabidopsis dicers 2, 3 and 4 [15]. We aimed to understand if the plant dicers functioned similarly in the *N. attenuata*- *M. sexta* model. To select the most effective combination of DCLs to silence, so as to increase the concentration of the retained longer dsRNA, we *Agro*-infiltrated all 16 combinations of the four *Na*DCL constructs into the stable PMRi line *ir-*CYP6B46 (30-2) (Fig. 4A). We found that silencing *Na*DCL 4, or the simultaneous silencing of any three *Na*DCLs significantly increased the accumulation of long dsRNA (102 bp that could be quantified by qRT-PCR) in plant leaves (*p≤*0.05; Fig. 4B, S4A). We selected the combinations of *Na*DCL 1, 3 and 4, and *Na*DCL 2, 3 and 4 for further experiments, based on the low variance as indicated by smaller standard errors of the long dsRNA transcript abundance mean values, as well as based on the previous PMRi report of the Arabidopsis *dcl* triple mutant [15]. Silencing of each *Na*DCL by the *Na*DCL 1, 3 and 4, or *Na*DCL 2, 3 and 4 VIGS construct combinations was confirmed by transcript quantification (Fig S4B- S4E). *M. sexta* larvae were fed *ir*-CYP6B46 (30-2) plants infiltrated with these *Na*DCL VIGS construct combinations. WT as well as *ir*-CYP6B46 (30-2) plants infiltrated with empty VIGS vector (EV) were used as controls. After feeding on the two combination *Na*DCL VIGS plants for 14 d, larval midguts showed 50% reductions in CYP6B46 transcripts as compared to those of larvae fed on *ir*-CYP6B46 (30-2)-EV plants (*p*≤0.05) (Fig. 4C), demonstrating that the silencing the plant's dicer machinery had increased the silencing efficiency of the PMRi by a factor of 2.

## Discussion

PMRi has the potential of becoming a crop protection tool targeting insect pests with far greater specificity than currently available pesticides or xenobiotics such as the *Bt* toxin [15]. Especially in the case of lepidopteran insects, CYPs that detoxify plant defense compounds or synthetic insecticides are potential targets of RNAi. Silencing these CYPs, suppresses transcript levels of the targeted genes, attenuating their function, and influencing larval growth or survival [15], [17], [33]. Since they belong to multigene families, CYPs have also been proposed to be the ideal targets for combinatorial RNAi [17]. Therefore in this first attempt of insect gene silencing by transient plant viral system, we considered CYPs as valuable candidate genes.

Snyder and colleagues demonstrated that CYP4M1 and CYP4M3 were upregulated in *M. sexta* larval midgut in response to nicotine ingestion [31]. Similarly in a microarray analysis, Govind and colleagues showed that CYP6B46 was downregulated in the larval midgut, when fed on nicotine suppressed *ir*-PMT plants [30]. Based on their response to dietary nicotine, we selected these three genes (CYP6B46, CYP4M1 and CYP4M3) for the PMRi trial. *M. sexta* is the specialist herbivore of host plants that produce high concentrations of nicotine and is famous for having the highest tolerance to nicotine of any organism [34].

Several researchers demonstrated a successful insect gene silencing with the *Agrobacterium*-transformed stable or transient transgenic plant mediated RNAi [13], [14], [15], [17]. We report similar silencing for the midgut-based CYP6B46 of *M. sexta*, suggesting that the PMRi is a reproducible and robust technique. Additionally, we showed that the silencing was highly specific and did not spread even to the most similar genes of the same family. Such specificity and reproducibility was important to demonstrate, as the RNAi in Lepidoptera has recently been suggested to be dependent on the insect, gene, gene function, organ of expression and mode of delivery [2], [24]. More importantly for our aims, this work demonstrates that the *M. sexta*- *N. attenuata* ecological model was amenable to PMRi.

Successful PMRi in *M. sexta* allowed us to use it as benchmark for developing an easier and faster VDPS method. While trying the VDPS that was previously successful against root nematodes [26], [27], we found that the silencing efficiency of VDPS and PMRi was similar. The VDPS experiments could be accomplished within three months in contrast to the PMRi procedure that requires a year of laborious screening of plants through two generations. Since VDPS is a more rapid technique, we could screen three CYPs in a short time and found CYP4M3 to be good candidate for further research. Since the larval growth was reduced while feeding on nicotine containing VDPS-CYP4M3 plants (Fig. 3J), this gene among the three CYPs tested, may play a central role in increasing *M. sexta*'s tolerance to nicotine ingestion.

Similar to stable PMRi, VDPS was also found to be highly specific in its silencing of all three candidate genes. PMRi is clearly the method of choice for crop protection in countries which allow the growth of transgenic crops and could be of immediate utility in the control of polyphagous pests such as *H. armigera*. However, VDPS could be a method of choice for high throughput reverse genetic screens of potential genetic targets in insect pests, as well as enabling research into a multitude of unanswered ecological questions at the molecular level.

The length of dsRNA is often of concern in RNAi experiments. In most of the experiments on insects that were based on the trophic delivery of dsRNA, different lengths of dsRNA (300–520 bp) has been used [2]. In the TRV based VDPS used against nematodes, inserts greater than 150 bp were recommended [27]. However, cloning of small sense or antisense fragments and especially small hairpins were also shown to be effective in the TRV vectors [27], [35]. Therefore the standardization of insert length would be an important consideration for the future VDPS experiments. We addressed the issue of dsRNA length in the PMRi experiment. Silencing of CYP6B46 was enhanced after increasing the length of ingested dsRNA by silencing the plant's dicers. These results were consistent with the findings in *H. armigera* [15] and are consistent with the hypothesis that the efficiency of RNAi depends on the length of the ingested dsRNA. Recent discoveries have shown that the dsRNase from *B. mori* midgut is synthesized in midgut cells and subsequently secreted into the lumen [36], [37], [38]. Thus, it is possible that the lepidopteran dicers that function in extremely alkaline environments of the midgut are specialized and possess different dicing properties than do the plant dicers; consequently, insect-dicer diced smRNA might be more effective than the plant-dicer diced smRNA in gene silencing in insects.

We conclude that stable PMRi can be a specific and robust system of gene silencing in *M. sexta*. While retaining all the virtues of PMRi, VDPS promises to be a rapid and high throughput alternative, suitable for ecological research. Silencing of plant dicers in PMRi lines revealed that similar to the results obtained in *H. armigera*, the gene silencing effect is enhanced in *M. sexta* suggesting that plant and insect RNAi machinery respond differently to the varying lengths of dsRNA.

## Materials and Methods

### Plant material

*N. attenuata* 30× inbred seeds, which were originally collected in 1988 from a native population at Utah (United States) were used for the generation of *Agrobacterium tumefaciens* mediated stable transgenic as well as VDPS lines in all the experiments. The seeds were germinated on sterile Gamborg B5 medium (Sigma, Germany) after 1 h of treatment with 50× (V/V) diluted smoke (House of Herbs) and 1 µM GA_3_. Ten days after germination, seedlings were transferred to Teku pots containing peat-based substrate, and after an additional 10 to 12 d, the plantlets were transplanted into individual 1L pots with the same substrate. In the glasshouse, plants were grown at 24°C to 26°C, relative humidity approximately 55%, and supplemented with light from 400- and 600-W sodium lamps (Philips) for 16 h [39].

### Insect culture

Eggs from an in-house *M. sexta* colony were stored in a growth chamber (Snijders Scientific) at 26°C- 16 h light, 24°C- 8 h darkness and 65% relative humidity, until the larvae hatched. Three freshly hatched neonates were placed on each rosette-stage *N. attenuata* plant. Fourteen day old fourth instar larvae were used in all assays, unless specified otherwise.

### Analysis of *Ms*CYP sequences and their selection for cloning in PMRi or VDPS vector

Sequences of 23 *M. sexta* CYPs were retrieved as complete coding sequences from NCBI. Their ORFs were aligned and a phylogenetic tree was constructed using Clustal W, with 1000 bootstrapping trials, to identify the most closely related *Ms*CYPs to the preselected targets of gene silencing: CYP6B46 (GU731529), CYP4M1 (GU731525) and CYP4M3 (GU731527).

Sequences of candidate gene cDNA fragments (≥300 bp) to be cloned in the PMRi or VDPS vector were selected based on two criteria: 1) the availability of primer binding sites ≥300 bp apart from each other in the candidate cDNA, according to the ‘Primer3’ online utility [40] and 2) the degree of similarity of this ≥300 bp fragment with their homologous region in the allied genes. This was considered in order to avoid co-silencing of closely related genes due to their high sequence similarity and to enable detection of ‘off-target’ effects of gene silencing [24]. Thus, CYP6B46 fragment was aligned with its homolog from CYP6B45 (GU731528), and the CYP4M1 and CYP4M3 fragments (which are already highly homologous to each other) were aligned with their closest homolog, CYP4M2 (L38671) (Fig. S1A, S1B).

### Larval tissues

Hemolymph, Malpighian tubules, fat body, foregut, midgut and hindgut were collected from the fifth instar larvae for transcript profiling of the selected *Ms*CYPs. Hemolymph of each larva was collected separately by clipping the horn. Then, these larvae were dissected in 0.15 M NaCl under a dissecting microscope. The 14 d old fourth instar larvae used in all gene silencing assays were also dissected in 0.15 M NaCl to isolate their midguts. Dissected gut tissues were carefully washed in 0.15 M NaCl to remove any adhering plant material. All tissues were stored in TRI regent (Invitrogen) as recommended by the manufacturer, until further use.

### Total RNA isolation

Total RNA was extracted from TRI regent stored tissues using manufacturer's protocol. Isolated total RNA was always subjected to TURBO DNase (Ambion) treatment, according to the manufacturer's protocol.

### Real time quantitative PCR

All the primer sequences used for real time quantitative PCR (qRT-PCR) are listed in Table S1. These primers were designed from the transcript sequences retrieved from NCBI, using Primer3 [40], to amplify ≥100 bp in the respective template cDNA, upstream of the selected ≥300 bp regions. This upstream position was targeted because, in the organisms such as plants, nematodes and fruit flies in which the RNAi pathway has been well characterized, silencing is known to spread in a 3′ to 5′ direction of the targeted mRNA [3]; hence the 5′ region of the target mRNA is longer lived and more available for qRT-PCR based quantification than regions in the 3′ region. For CYP6B46, CYP6B45, CYP4M1, CYP4M2 and CYPM3, the size of this amplicon was 118BP (+144 to +261), 105 bp (+613 to +718), 101 bp (+868 to +968), 166 bp (+453 to +619) and 130 bp (+703 to +832), respectively. The specificity of these primers for insect cDNA was confirmed by a PCR with *N. attenuata* (WT) leaf cDNA.

For each sample, 500 ng of total RNA was used for cDNA preparation using oligo(dT)**_18_** primer and SuperScript II enzyme (Invitrogen) with the manufacturer's recommendations. All the qRT-PCRs were performed with a Mx3005P Multiplex qPCR system (Stratagene) and the qPCR core kit for SYBR Green I (Eurogentec). Relative quantification of mRNA levels was performed by the comparative Δ cycle threshold (CT) method using the *Ms*Ubiquitin mRNA as an internal standard. All the qPCRs were performed using the following conditions: initial denaturation step of 95°C for 30 s, followed by 40 cycles each of 95°C for 30 s and 60°C for 1 min, with a final extension step of 95°C for 30 s and 60°C for 1 min. All the results were obtained from at least five independent biological replicates and two technical replicates.

### Plant transformation

Stable transgenic inverted repeat-*Ms*CYP6B46 (*ir-*CYP6B46) *N. attenuata* lines were generated by transforming the recombinant pSOL8 transformation vector [28]. Vector contained 300 bp fragment of *M. sexta* CYP6B46 gene in an inverted repeat orientation along with the hygromycin phosphotransferase (*hptII*) gene providing hygromycin resistance as a selectable marker (Fig. S2A) [41]. Screening of the transgenic lines followed the protocol recommended by Gase *et al.* [42]. Two homozygous independently transformed *ir-*CYP6B46 lines (30-2 and 416-3) harboring single insertions of the *hptII* marker gene were used for further studies. Wild type (WT) and the stable transgenic empty pSOL8 vector (EV) containing [43] plants were used as negative controls.

### Southern Hybridization

A modified cetyltrimethylammonium bromide method described by Rogers and Bendich [44] was followed for genomic DNA extraction from the fully expanded rosette leaves of WT, *ir-*CYP6B46 (30-2) and *ir-*CYP6B46 (416-3) *N. attenuata* plants. For Southern-blot hybridizations 10 µg of the genomic DNA was completely digested with *HindIII* and *EcoRV*, separately. It was then size fractionated on 1% (w/v) agarose gel, and blotted onto a nylon membrane (GeneScreenPlus; Perkin-Elmer) by capillary transfer. Hybridization and detection of the insertion number was performed as described by Gase *et al*. [42].

### Virus induced plant gene silencing (VIGS)

A VIGS system based on the TRV was used to silence *N. attenuata* DCLs, as described by Saedler and Baldwin [29]. VIGS vector (pTV) harboring ≥300 bp fragments of *Na*DCL1 (JN032013), *Na-*DCL2 (JN032015), *Na*DCL3 (JN032015), and *Na*DCL4 (JN032016) (Bozorov *et al*., in review) were used for VIGS. A vector without insert (EV) was used as a negative control. To silence the four *Na*DCLs in various combinations, the *Agrobacterium* cultures containing respective DCL-VIGS constructs were mixed in equal proportions before infiltration in three *ir-*CYP6B46 (30-2) plants. Primers were designed to amplify 102 bp of the cloned and expressed *Ms*CYP6B46 dsRNA in leaf (Fig. S4A; Table S1). Plants with DCL-VIGS combination retaining higher levels of 102 bp *Ms*CYP6B46 transcripts were detected by qRT-PCR and were selected for feeding *M. sexta* larvae. Silencing of each *Na*DCL used in the selected VIGS combinations was confirmed by the qRT-PCR based transcript quantification (Fig S4B- S4E). Prior to this work, it was confirmed that the primers used for *Na*DCL transcript quantification (Table S1) produced a single amplicon with *N. attenuata* (WT) cDNA (Fig S4F).

### Plant tissue, RNA isolation, cDNA synthesis and qRT-PCR

Fully expanded *N. attenuata* rosette leaves were used in the entire analysis. They were harvested and immediately frozen in liquid nitrogen. The protocols used for insect tissues were also followed for their RNA isolation, cDNA synthesis and qRT-PCR. *Na*Actin was used as an internal standard for qRT-PCR. All the results were obtained from three independent biological replicates and two technical replicates.

### Plant-Virus based dsRNA Producing System (VDPS)

*N. attenuata* VIGS system was modified with the substitution of inserted plant cDNA fragment by the insect cDNA fragment. Selected ≥300 bp fragment of *Ms*CYP6B46 or *Ms*CYP4M1 or *Ms*CYP4M3 was cloned in the pTV vector in an antisense orientation (Fig. S3) and *Agro*-infiltrated into WT plants to express ≥300 bp dsRNA of the respective cloned fragment. An empty pTV vector (EV) was used as a negative control. These transformed plants were fed to *M. sexta* larvae to silence the expression of *Ms*CYP6B46, *Ms*CYP4M1 and *Ms*CYP4M3, respectively.

### Low Molecular Weight (LMW) RNA isolation

For the isolation of low molecular weight (LMW) RNA, the total RNA (from insect midgut as well as from leaves) was subjected to PEG (10%) precipitation in presence of 1 M NaCl. Subsequently, it was precipitated using isopropyl alcohol (0.8 volumes) by incubating overnight at −20°C.

### Northern blotting and hybridization for the detection of smRNA

Fifty µg LMW RNA (pool of three biological replicates from insect midguts or leaves) was separated under denaturing conditions (8 M urea) in 15% acrylamide gel, stained with ethidium bromide and visualized with the help of a UV transilluminator. Similar fluorescence intensity of the 5.8 S rRNA bands reflected the equal loading of LMW RNA. The RNA was transferred to a nylon membrane (GeneScreenPlus; Perkin-Elmer) by electro-blotting at 400 mA for 1 h, using 0.5× TBE as a transfer buffer. Fragments of *Ms*CYP6B46 were PCR amplified using primer pair #4 (Table S1) for the preparation of the probe. Ten ng of these amplicons were labeled with α-^32^P using the Rediprime II DNA labeling system (Amersham Biosciences). Hybridization and screening of the blot performed as previously explained, except that the blots were exposed to the Fujifilm (www.fujifilm.com) BAS-MS imaging plates for 10 to 12 days until no further increase in signal was observed [45].

### Statistical analysis

All statistical analyses were performed with StatView version 5 (SAS Institute Inc.). Significance of variance was determined after the one way ANOVA (P>0.05) and was represented in all the graphs as ±S.E.

## Supporting Information

Figure S1 **Alignments of** ***Ms*** **CYPs cDNA regions selected for cloning; insect cDNA specific amplification by qRT-PCR primers.** Sequence alignments of *M. sexta* CYP cDNA fragments that were selected for PMRi experiments, with the homologous sequences of respective allied genes that were tested for off-target co-silencing effects (see Fig. 1A) (**A**) Alignment of the selected CYP6B46 cDNA fragment with the homologous fragment from the CYP6B45 cDNA. (**B**) Alignment of selected cDNA fragments of CYP4M1 and CYP4M3 with each other and with the homologous cDNA fragment from CYP4M2. (**C**) RT-PCR analysis showing that the primers used for the SYBR-Green qRT-PCR produced single amplicons (resolved on 2% agarose gel) with *M. sexta* cDNA and did not produce amplicons with *N. attenuata* leaf cDNA, demonstrating that the primers were insect cDNA specific. A 100 bp ladder was used as a size marker.
(TIF)

Figure S2 **Map of plant stable transformation vector and detection of a single transgene insertion in the transformed lines.** (**A**) A map of pSOL8 vector used for *Agrobacterium tumefaciens* mediated plant transformation harboring the inverted repeat (separated by the *pdk i3* intron) of a 312 bp fragment of *M. sexta*'s CYP6B46 cDNA. (**B**) Southern hybridization after *HindIII* digested genomic DNA, showing the presence of a single insertion of transgene in both the independently transformed (30-2 and 416-3) *N. attenuata* lines; WT control shows absence of transgene insertion. 1 kb DNA ladder was used as a size marker.
(TIF)

Figure S3 **Map of viral dsRNA-producing system (VDPS) vector.** A map of tobacco rattle virus (TRV) based plant (transient) transformation vector pTV. A ≥300 bp stretch from *M. sexta*'s CYP6B46/CYP4M1/CYP4M3 cDNA was cloned in an antisense orientation into pTV for VDPS. *N. attenuata* (WT) plants were transiently transformed with this recombinant vector by *Agro*-infiltration and *M. sexta* larvae were fed on the leaves of inoculated plants to silence the expression of CYP6B46/CYP4M1/CYP4M3 genes, respectively, in their midguts.
(TIF)

Figure S4 **Schematic representation of cDNA regions used for** ***Ms*** **CYP6B46 transcript profiling in leaves and larval midguts.** For transcript quantification in plant material, a 102 bp fragment residing inside the 312 bp region (300 to 612 b of the ORF) that was cloned into the pSOL8 vector in an inverted repeat orientation was used. After silencing plant's four DCL genes, the abundance of the transcripts of this fragment increased. Since this fragment was longer than the diced transcripts (21–24 bp), it provided a measure of the abundance of undiced transcripts. For the quantification of transcripts in larval midguts, another100 bp fragment, located 5′ of the cloned region was used. Using a fragment outside the cloned region ensured that the quantification of endogenous gene silencing would not be confounded by undiced dsRNA or vector-born transcripts. Transcript abundance of *N. attenuata*'s (**B**) DCL1, (**C**) DCL2, (**D**) DCL3 and (**E**) DCL4 in the leaves of WT *Agro*-infiltrated with EV, *ir-*CYP6B46 (30-2) *Agro*-infiltrated with EV or DCL1, 3 and 4 or DCL2, 3 and 4, respectively. (**F**) RT-PCR analysis showing that the *Na*DCL and *Na*Actin primers used for the qRT-PCR, produced single amplicons (resolved on 2% agarose gel) with *N. attenuata* (WT) cDNA. A 100 bp ladder was used as a size marker. Bars labeled with different letters indicate significant differences as determined by one way ANOVAs (*p*≤0.05).
(TIF)

Table S1 ***M. sexta*** **and** ***N. attenuata*** **gene primers.**
(DOC)
