# Supplementary figures and images for: Tobacco Rattle Virus Vector: A Rapid and Transient Means of Silencing Manduca sexta Genes by Plant Mediated RNA Interference
Source: PLoS One. 2012 Feb 1;7(2):e31347. doi: 10.1371/journal.pone.0031347 (PMC3270032; doi:10.1371/journal.pone.0031347)

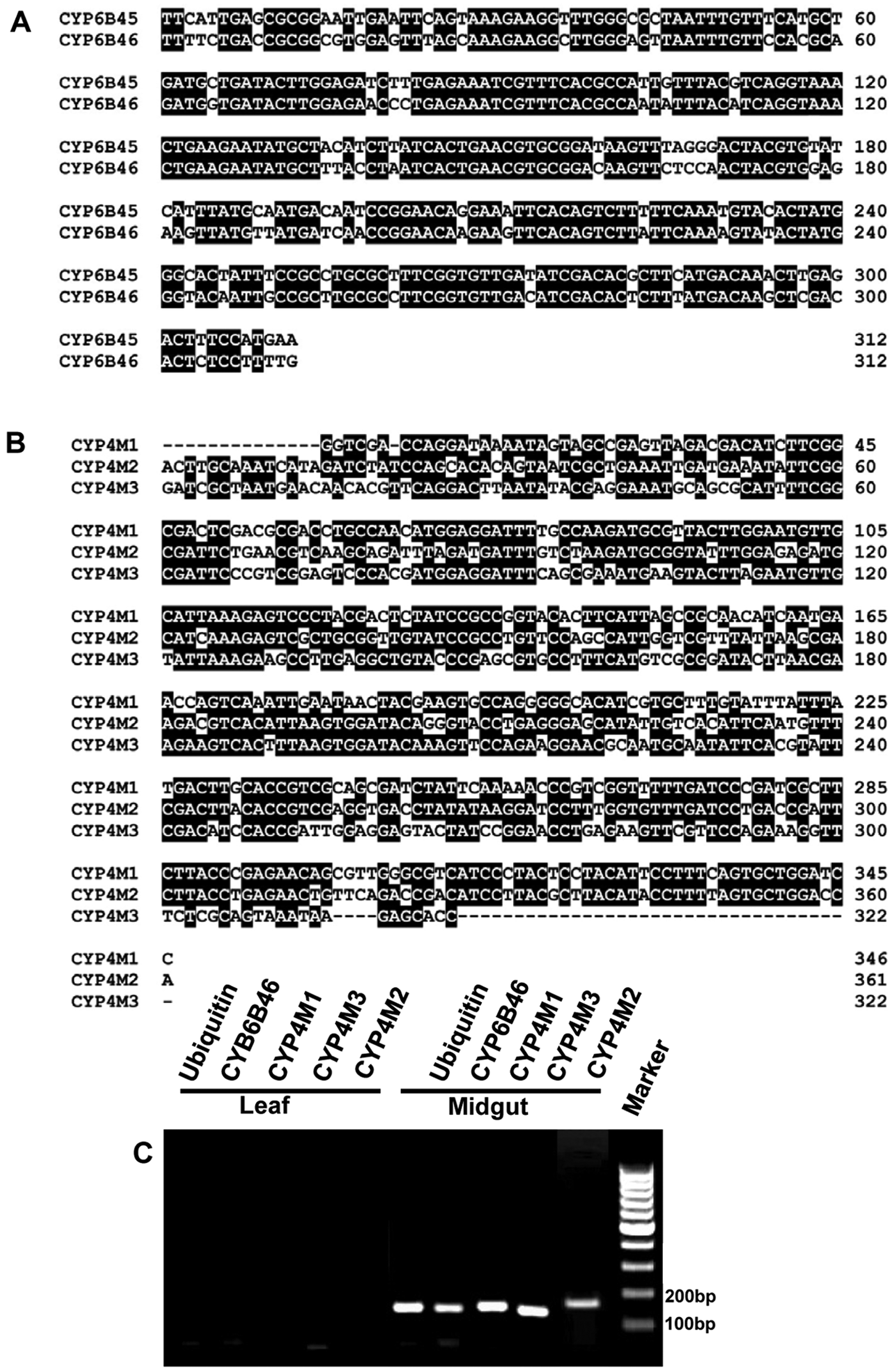

Supplement: Figure S1 — Alignments of Ms CYPs cDNA regions selected for cloning; insect cDNA specific amplification by qRT-PCR primers. Sequence alignments of M. sexta CYP cDNA fragments that were selected for PMRi experiments, with the homologous sequences of respective allied genes that were tested for off-target co-silencing effects (see Fig. 1A) (A) Alignment of the selected CYP6B46 cDNA fragment with the homologous fragment from the CYP6B45 cDNA. (B) Alignment of selected cDNA fragments of CYP4M1 and CYP4M3 with each other and with the homologous cDNA fragment from CYP4M2. (C) RT-PCR analysis showing that the primers used for the SYBR-Green qRT-PCR produced single amplicons (resolved on 2% agarose gel) with M. sexta cDNA and did not produce amplicons with N. attenuata leaf cDNA, demonstrating that the primers were insect cDNA specific. A 100 bp ladder was used as a size marker. (TIF) [file pone.0031347.s001.tif]

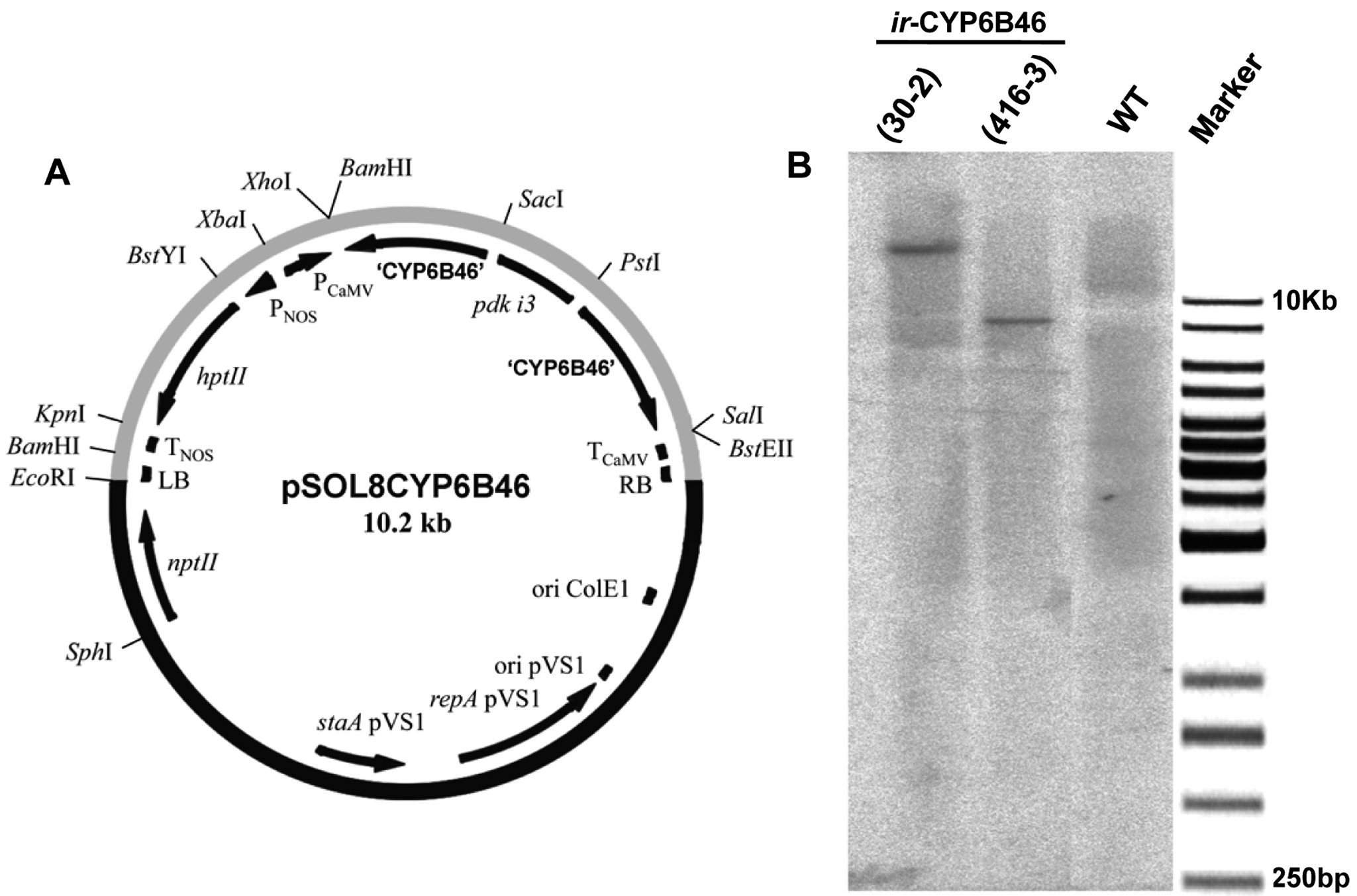

Supplement: Figure S2 — Map of plant stable transformation vector and detection of a single transgene insertion in the transformed lines. (A) A map of pSOL8 vector used for Agrobacterium tumefaciens mediated plant transformation harboring the inverted repeat (separated by the pdk i3 intron) of a 312 bp fragment of M. sexta's CYP6B46 cDNA. (B) Southern hybridization after HindIII digested genomic DNA, showing the presence of a single insertion of transgene in both the independently transformed (30-2 and 416-3) N. attenuata lines; WT control shows absence of transgene insertion. 1 kb DNA ladder was used as a size marker. (TIF) [file pone.0031347.s002.tif]

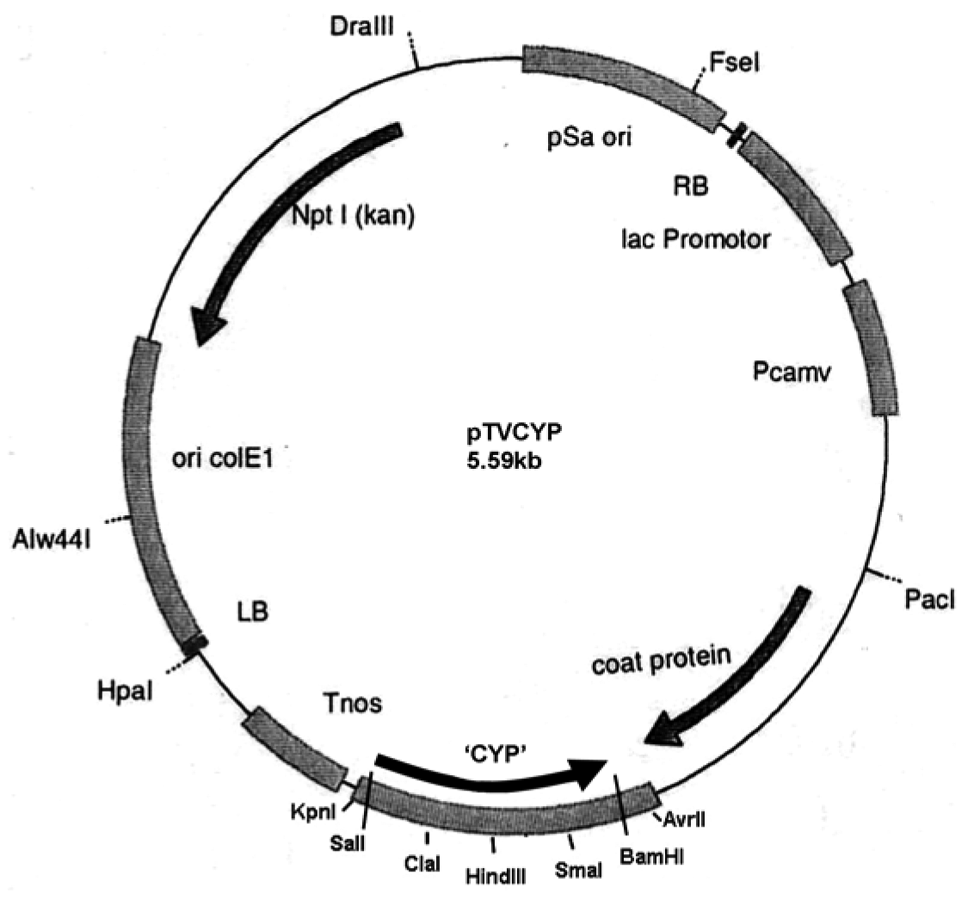

Supplement: Figure S3 — Map of viral dsRNA-producing system (VDPS) vector. A map of tobacco rattle virus (TRV) based plant (transient) transformation vector pTV. A ≥300 bp stretch from M. sexta's CYP6B46/CYP4M1/CYP4M3 cDNA was cloned in an antisense orientation into pTV for VDPS. N. attenuata (WT) plants were transiently transformed with this recombinant vector by Agro-infiltration and M. sexta larvae were fed on the leaves of inoculated plants to silence the expression of CYP6B46/CYP4M1/CYP4M3 genes, respectively, in their midguts. (TIF) [file pone.0031347.s003.tif]

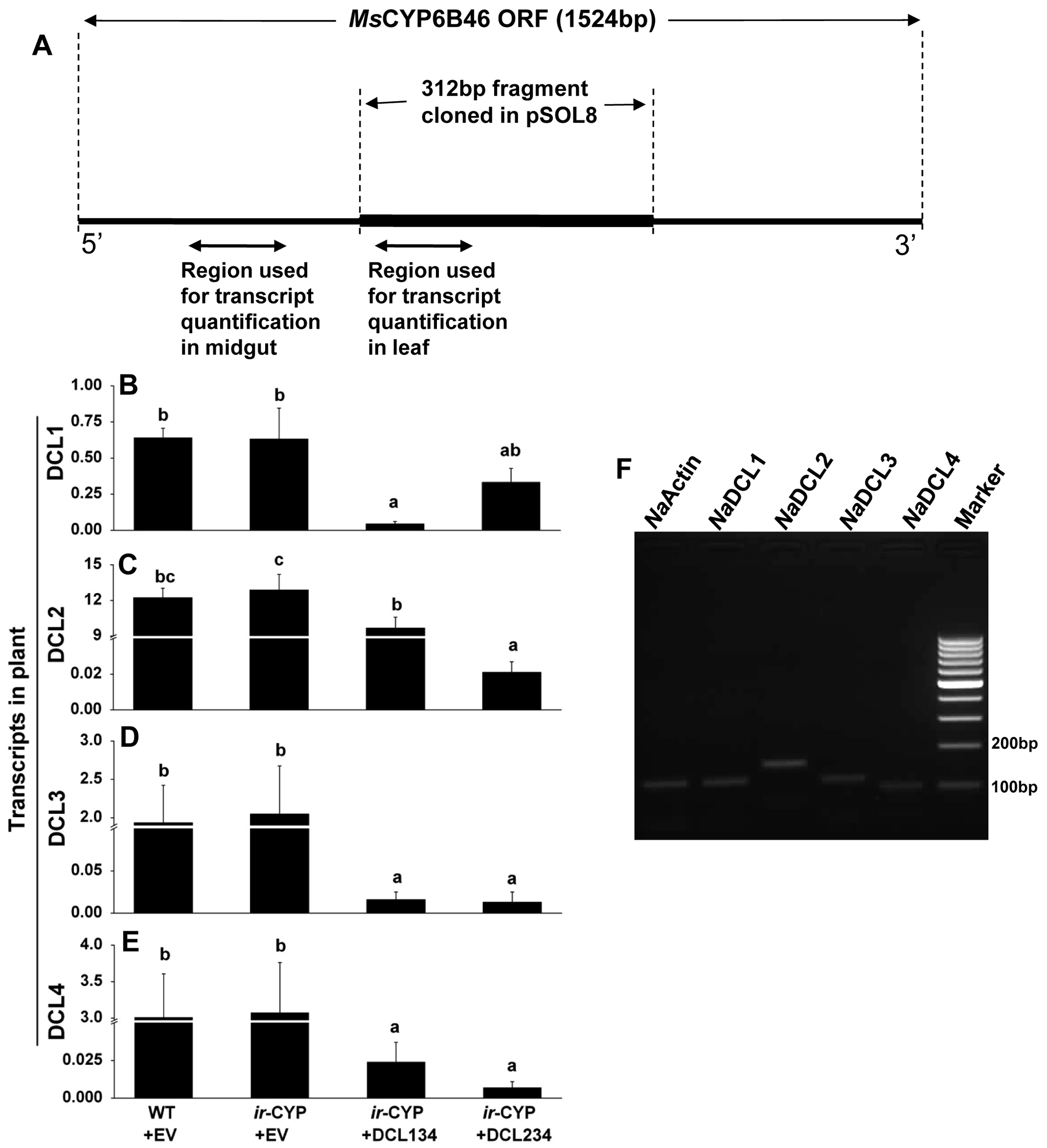

Supplement: Figure S4 — Schematic representation of cDNA regions used for Ms CYP6B46 transcript profiling in leaves and larval midguts. For transcript quantification in plant material, a 102 bp fragment residing inside the 312 bp region (300 to 612 b of the ORF) that was cloned into the pSOL8 vector in an inverted repeat orientation was used. After silencing plant's four DCL genes, the abundance of the transcripts of this fragment increased. Since this fragment was longer than the diced transcripts (21–24 bp), it provided a measure of the abundance of undiced transcripts. For the quantification of transcripts in larval midguts, another100 bp fragment, located 5′ of the cloned region was used. Using a fragment outside the cloned region ensured that the quantification of endogenous gene silencing would not be confounded by undiced dsRNA or vector-born transcripts. Transcript abundance of N. attenuata's (B) DCL1, (C) DCL2, (D) DCL3 and (E) DCL4 in the leaves of WT Agro-infiltrated with EV, ir-CYP6B46 (30-2) Agro-infiltrated with EV or DCL1, 3 and 4 or DCL2, 3 and 4, respectively. (F) RT-PCR analysis showing that the NaDCL and NaActin primers used for the qRT-PCR, produced single amplicons (resolved on 2% agarose gel) with N. attenuata (WT) cDNA. A 100 bp ladder was used as a size marker. Bars labeled with different letters indicate significant differences as determined by one way ANOVAs (p≤0.05). (TIF) [file pone.0031347.s004.tif]
