# Supplementary material for: Tobacco Rattle Virus Vector: A Rapid and Transient Means of Silencing Manduca sexta Genes by Plant Mediated RNA Interference
Source: PLoS One. 2012 Feb 1;7(2):e31347. doi: 10.1371/journal.pone.0031347 (PMC3270032; doi:10.1371/journal.pone.0031347)
Supplement: Table S1 — M. sexta and N. attenuata gene primers. (DOC) [file pone.0031347.s005.doc]

**Table S1.** *M. sexta* and *N. attenuata* gene primers

| **Primer pair no.** | **Gene** | **Primer sequences (5'-3')** | **Use** |
| --- | --- | --- | --- |
| **1** | ***MsUbiquitin*** | **For-** AAAGCCAAGATTCAAGATAAG  **Rev-** TTGTAGTCGGATAGCGTGCG | Internal control for *M. sexta* transcript quantification |
| **2** | ***MsCYP6B46*** | **For-** GTGCCTATTACTCCGCGATCTA**Rev-** CCAAGCCTTCTTTGCTAAACTCC | Transcript quantification and silencing efficiency testing of *M. sexta* CYP6B46 |
| **3** | ***MsCYP6B46*** | **For-** GCAGATGGTGATACTTGGAGAA  **Rev-** GTCCGCACGTTCAGTGATTAG | Transcript quantification of CYP6B46 dsRNA in DCL/s silenced leaf |
| **4** | ***MsCYP6B46*** | **For-** TTTTCTGACCGCGGCGTGG  **Rev-** AAAAGGAGAGTGTCGAGCTTG | Radiolabelled probe making for detection of *Ms*CYP6B46 small RNA |
| **5** | ***MsCYP4M1*** | **For-** GCTGAAAGAGATGGGGAAATC  **Rev-** CAAAACGTCAACCCAGAAGC | Transcript quantification and silencing efficiency testing of *M. sexta* CYP4M1 |
| **6** | ***MsCYP4M1*** | **For-** CAGGATAAAATAGTAGCCGAG  **Rev-** CAGCACTGAAGGGAATGTAG | Radiolabelled probe making for detection of *Ms*CYP4M1 small RNA |
| **7** | ***MsCYP4M2*** | **For-** GGTGCAGAATGTCGGCAAATC  **Rev-** TCTTCCGAGTGCGCAGATAG | Transcript quantification and co-silencing efficiency testing of *M. sexta* CYP4M2 |
| **8** | ***MsCYP4M3*** | **For-** AGACGTGCAGTCAAAGACCTG  **Rev-** CCATCCGACTTTTCTTACCG | Transcript quantification and silencing efficiency testing of *M. sexta* CYP4M3 |
| **9** | ***MsCYP4M3*** | **For-** GATCGCTAATGAACAACACGT  **Rev-** GGTGCTCTTATTTTCTGCGA | Radiolabelled probe making for detection of *Ms*CYP4M3 small RNA |
| **10** | ***MsCYP6B45*** | **For-** GAAATGGATAAATTGGTTTTGACC  **Rev-** TTATTTTGACAGAGAAGATTGAGG | Transcript quantification and co-silencing efficiency testing of *M. sexta* CYP6B45 |
| **11** | ***NaActin*** | **For-** GGTCGTACCACCGGTATTGT  **Rev-** GTCAAGACGGAGAATGGCAT | Internal control for *N. attenuata* transcript quantification |
| **12** | ***HptII*** | **For-** CGTCTGTCGAGAAGTTTCTG  **Rev-** CCGGATCGGACGATTGCG | Radiolabelled probe making for detection of transgene inserts in *N. attenuata* |
| **13** | ***NaDCL1*** | **For-** CCAGGCACAGGGAATTTTATC  **Rev-** AGGTGAACCAACTTTGAGCTG | Silencing efficiency testing of *N. attenuata* DCL1 |
| **14** | ***NaDCL2*** | **For-** CGATGAAGAATTGCTGATGC  **Rev-** GCCTGAGCTGAGAAAGGCAC | Silencing efficiency testing of *N. attenuata* DCL2 |
| **15** | ***NaDCL3*** | **For-** GCACTGATGGTGACATCTGC  **Rev-** CACTGCTAGGTTGAGCTTTGG | Silencing efficiency testing of *N. attenuata* DCL3 |
| **16** | ***NaDCL4*** | **For-** ACCAAGTGCTGCAACTTCAC  **Rev-** TCCTTCTCTGGTTTCTGAACTG | Silencing efficiency testing of *N. attenuata* DCL4 |
